# The Preparation and Properties of Ultra-High-Performance Concrete with Aeolian Sand: A Lab Study on the Effect of the Curing Method

**DOI:** 10.3390/ma18092031

**Published:** 2025-04-29

**Authors:** Yang Lv, Boyu Zhao, Jie Zhu, Chenhao He, Yunlu Ge, Yuanshuai Wu, Yanchao Zhu, Jianming Dan, Yang Zhou, Xiangguo Li

**Affiliations:** 1 State Key Laboratory of Silicate Materials for Architectures, Wuhan University of Technology, Wuhan 430070, China; yang.lv@whut.edu.cn (Y.L.); 331481@whut.edu.cn (B.Z.); hch@whut.edu.cn (C.H.); geyunlu0419@163.com (Y.G.); wuyuashuai@163.com (Y.W.); 2 Hubei Key Laboratory of Polymer Materials, Hubei University, Wuhan 430070, China; zhuyc@hubu.edu.cn; 3 China Railway Electrification Bureau Group Co., Ltd., Beijing 100071, China; 15347075977@163.com; 4 State Key Laboratory Incubation Base for Green Processing of Chemical Engineering, School of Chemistry and Chemical Engineering, Shihezi University, Shihezi 832003, China; danjmxj@163.com (J.D.); zhougroup@shzu.edu.cn (Y.Z.)

**Keywords:** ultra-high-performance concrete (UHPC), aeolian sand, mechanical properties, microstructure

## Abstract

The utilization of aeolian sand (AS) as a substitute for river sand (RS) in ultra-high-performance concrete (UHPC) offers a sustainable solution to address natural sand resource shortages while enhancing AS utilization. This study systematically evaluates the influence of AS content (0–100% RS replacement by mass) on the workability, mechanical properties, and microstructure of UHPC under different curing regimes. All mixtures incorporate 0.65% by volume of straight steel fibers to ensure adequate fiber reinforcement. The results reveal that the spherical morphology, smooth surface nature, and fine particle size of AS enhance the matrix fluidity and reduce the early autogenous shrinkage of UHPC. By employing steam curing at 90 °C for 2 d followed by standard curing for 7 d (M3), UHPC samples with a 60% and 80% AS substitution achieve a compressive strength of 132.4 MPa and 130.8 MPa, respectively; a flexural strength exceeding 18 MPa; a porosity below 10%; and a gel pore content exceeding 60%. The steel fiber reinforcement contributes significantly to the flexural performance, with the fiber–matrix interface quality maintained even at high AS replacement levels. These findings highlight the feasibility of AS as an alternative fine aggregate in UHPC.

## 1. Introduction

Ultra-high-performance concrete (UHPC) has attracted considerable interest in the construction industry owing to its outstanding strength, toughness, and durability [1,2,3]. UHPC typically incorporates fine aggregates, a high cementitious content, and low water-to-binder ratios, which enables a dense microstructure via optimized particle packing [4]. However, the rapid depletion of natural river sand (RS) necessitates sustainable alternatives, such as aeolian sand (AS), to mitigate the ecological damage caused by excessive mining [5]. Aeolian sand, primarily composed of quartz with minor feldspar and mica, originates from weathered sandstone and granite residues. Compared to RS, AS exhibits finer particle sizes (0.15–0.6 mm), a higher sphericity, and smoother surfaces [6,7].

Previous studies have explored the incorporation of AS in concrete. For workability, Al-Harthy et al. [8] and Amel et al. [9] observed a parabolic trend in slump with increasing AS content (20–60% optimal), attributing this to the spherical morphology of AS particles enhancing interparticle mobility. However, excessive AS (>60%) disrupts the aggregate gradation, increasing fine particle proportions and reducing slurry fluidity. Regarding the mechanical properties, Li et al. [10,11] demonstrated that moderate AS addition (≤60%) reduces porosity and enhances matrix densification through filler effects and heterogeneous nucleation. Reactive SiO_2_ in AS reacts with cementitious phases to form C-S-H/C-A-H gels, which bridge interfacial cracks and refine pore structures. Conversely, Luo et al. [12] emphasized that ultrafine AS particles (<175 μm) act as nucleation sites for hydration products, accelerating cement hydration. However, excessive AS weakens the interfacial transition zone (ITZ) by increasing harmful pores and microcracks, ultimately degrading strength. However, Al-Harthy et al. [4] and Seif et al. [13] obtained a different conclusion, noting that the compressive strength of concrete tends to decrease with the increase in the dosage of AS, which is due to the differences in the physical and chemical characteristics of AS in different regions. For example, the fineness modulus of AS from the Maowusu Desert region of China is much lower, its specific surface area is larger, its water absorption is higher, and its fluidity is poorer than that of the AS from West Asia and North Africa. This leads to a large difference in the properties of the concrete obtained from AS prepared with different physical and chemical characteristics.

The existing literature shows that AS can partially replace natural RS in concrete. However, the current substitution ratio of aggregates remains insufficient to effectively alleviate the shortage of natural RS resources. Additionally, conventional curing methods may degrade the performance of AS–concrete mixtures. Such degradation often manifests as reduced strength, compromised workability during construction, and diminished durability. These limitations make it difficult for AS–concrete to meet the practical requirements of structural engineering projects.

While AS demonstrates potential as a partial RS substitute, conventional curing methods often fail to optimize its performance in UHPC, resulting in inconsistent strength, durability, and workability. In response to the above issues, this study proposes an improved Andreas Andersen particle filling model to systematically evaluate the synergistic effects of the AS substitution rate (0–100%) and a multi-stage curing system (steam + standard curing) on UHPC performance. By combining thermogravimetric analysis, mercury intrusion porosimetry, and SEM, the mechanism of AS on hydration kinetics, pore evolution, and the fiber matrix interface is elucidated. This article aims to address the following key issues: (1) improving the substitution rate of AS while maintaining the mechanical properties of UHPC; (2) maintaining the dense microstructure of UHPC through gradation optimization under a high AS substitution rate; and (3) maintain the development of the basic performance of UHPC under a high AS substitution rate with the multi-stage curing system. The research results will provide basic theoretical support for the efficient utilization of AS resources, promote the transformation of UHPC towards low-carbon and low-cost directions, and have important practical significance for alleviating the shortage of natural sand and gravel resources and promoting the efficient utilization of local resources in desert areas.

## 2. Materials and Methods

### 2.1. Materials

This study adopts P O. 52.5 grade ordinary Portland cement provided by Shandong Qingyun Kangjing Building Materials Co., Ltd., in Dezhou, China. Polycarboxylate superplasticizer (SP) is used to regulate the workability of concrete. Commercial silica fume (SF) and blast furnace slag powder (Slag) are used as volcanic ash cementitious materials to partially replace cement. The silica fume is provided by Henan Zhengzhou New Materials Co., Ltd., (Zhengzhou, China). The blast furnace slag powder is the V800 ultrafine slag powder provided by Wuhan Huashen Intelligent Co., Ltd., (Wuhan, China). River sand with an original fineness modulus of 2.4 and a continuous particle size distribution is divided into two groups: one group is fine grain river sand with a particle size of 0–0.6 mm (RS 1), and the other group is coarse grain river sand with a particle size of 0.6–1.18 mm (RS 2). The particle size of AS is between 0 and 0.6 mm. The steel fibers used are long straight steel fibers (LSF) with a diameter of 0.2 mm, a length of 13 mm, and a tensile strength of 2929 MPa. Table 1 shows the chemical properties of the binder materials analyzed by X-ray fluorescence (XRF).

### 2.2. Experimental Methodology

#### 2.2.1. Mixture Ratio Design

The modified model based on the Andreasen and Andersen equations proposed by Funk and Dinger is the most widely used for UHPC at present [14]. The modified Andreasen–Andersen (A&A) model is shown in Equation (1) [15,16].

(1) $$P(D)=\frac{D^{q}-D_{min}^{q}}{D_{max}^{q}-D_{min}^{q}}$$

where P(D) denotes the total number of particles under the sieve (%); D denotes the current particle size (μm); D_min_ and D_max_ denote the minimum and maximum particle sizes of the particles (μm); and q is the distribution coefficient.

The modified Andreasen and Andersen infill model is now successfully applied in UHPC [17,18]. By adjusting the distribution coefficient q in Equation (1), it was possible to change the ratio and admixture between fine and coarse particles in the concrete particle system. The use of higher distribution coefficients (q > 0.5) led to an overall coarsening of the mixed particles, whereas lower distribution coefficients (q < 0.23) led to a particulate system enriched with more fine particles [19]. Brouwers [20] has demonstrated through several experiments that a distribution coefficient q value in the range of 0–0.28 produces an optimal system of stacked particles, and Hunger [21] suggested that distribution coefficient values in the range of 0.22–0.25 should be used in SCC design. Therefore, the q value was fixed at 0.23 considering that a large number of fine particles were used for the preparation of UHPC.

In this study, the modified Andreasen and Andersen model was used as an objective function to optimize the overall particle size ratio of the concrete system. The optimization algorithm based on the least squares method (LSM) was employed to adjust the admixture amounts of individual materials in the mixture. This adjustment process was integrated with actual production applications, resulting in an improved particle size distribution and optimized UHPC mix ratios. Furthermore, the algorithm facilitated precise fitting to the target curve of the mixture, which was calculated using the modified Andreasen and Andersen model as referenced in Equation (2) [22]. RSS denotes the residual difference sum of squares; the smaller the value, the smaller the error between the actual stacking curve and the target curve.

(2) $$RSS=\Sigma_{i=1}^{n}(P_{mix}(D_{i}^{i+1})-P_{tar}(D_{i}^{i+1}){)}^{2}$$

where P_mix_ is the composed mixture material and P_tar_ is the target gradation calculated according to Equation (1).

**Figure 1:**
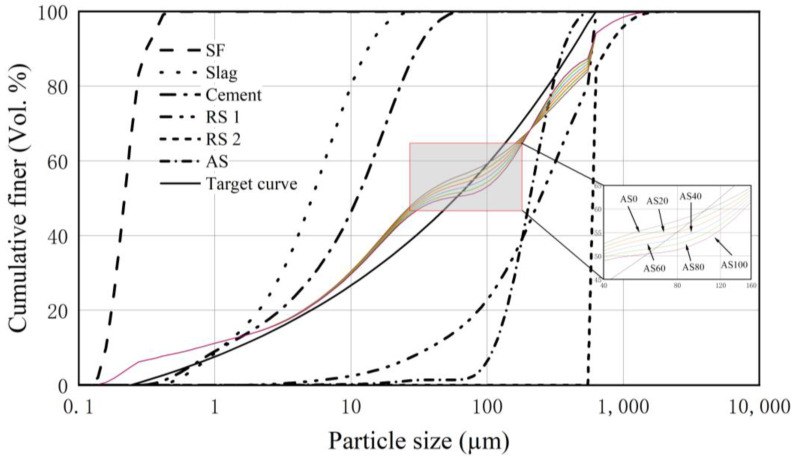
Particle accumulation curves of UHPC raw materials.

Based on the optimized particle filling model, the mixing ratio of the cementitious materials (cement, SF, and Slag) and the fine aggregate (RS 1, RS 2, and AS) particles was adjusted in conjunction with their particle size distributions so that the actual stacking state between the component particles of the designed UHPC was close to the most compact stacking state. The fits of UHPC formulated by replacing RS 1 with different proportions of AS in an equal mass with the same grain size distribution are shown in Table 2, and the stacking curves are shown in Figure 1. The results show that the particle gradation of AS in the same particle size range is not exactly the same as that of RS, but the actual stacking curves between the component particles are in good agreement with the target curves, which indicates that the stacking forms between the component particles of UHPC mixed with AS have not been significantly damaged, and the matrix is still in a state of compact stacking.

The mixture proportions for all UHPC formulations are presented in Table 2. The acronyms UHPC-1 through UHPC-6 represent six distinct concrete mixtures with varying AS replacement levels (0–100% of RS 1 by mass). Each mixture designation corresponds to a unique composition that was tested as a complete set of specimens for all experimental measurements. For each mixture (UHPC-1-UHPC-6), multiple specimens were prepared and tested under each curing regime to ensure the statistical reliability of the results. The number of replicates for each test is provided in the respective Experimental Methods sections.

Preliminary trials identified 60–80% AS substitution as critical thresholds where mechanical performance (e.g., compressive strength > 120 MPa) remained compliant with UHPC standards while maximizing AS utilization. Consequently, UHPC-4 (60% AS) and UHPC-5 (80% AS) were prioritized for microstructure and pore structure analysis to investigate the interplay between high AS content and optimized curing regimes.

#### 2.2.2. Mixing Procedures and Curing Methods

**Figure 2:**
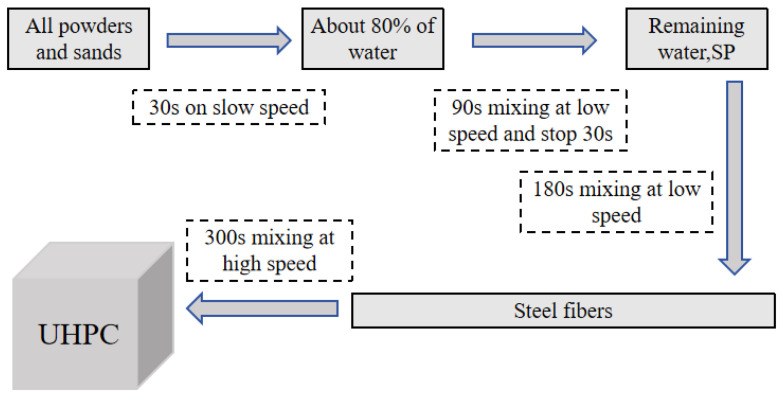
UHPC production process.

**Figure 3:**
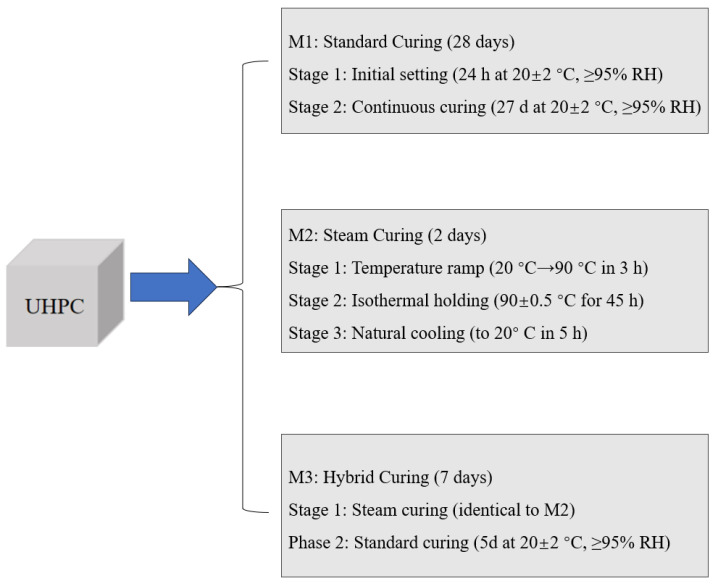
UHPC curing procedure flowchart.

The mixing procedure, as shown in Figure 2, was followed for UHPC preparation. After mixing, the prepared UHPC specimens were cured using different curing conditions, namely: (1) M1: Standard curing (20 ± 2 °C, ≥95% RH, 28 d): This represented the baseline condition and allowed for comparison with conventional UHPC curing practices. The extended 28 d duration enabled the complete evaluation of long-term hydration and strength development under normal conditions. (2) M2: Steam curing (90 °C, ≥95% RH, 2 d): This accelerated curing regime was chosen because high-temperature steam curing is commonly used in precast UHPC production to achieve early-age strength. The 2 d duration represented the typical industrial practice for thermal activation. The temperature of 90 °C was selected as it optimally activated pozzolanic reactions without causing detrimental effects to the microstructure. (3) M3: Combined curing (steam, 90 °C, 2 d + standard curing 5 d): This hybrid approach was designed to evaluate the effects of continued hydration after initial steam curing. Practical scenarios where steam-cured elements experienced subsequent ambient conditions were simulated. The 7 d total duration allowed for the direct comparison with standard test ages while capturing the critical early-age development. The specific sample curing process diagram is shown in Figure 3. Table 3 shows the annotations and meanings of each acronym.

These regimes were selected based on preliminary studies and industry standards to provide comprehensive data on AS-UHPC behavior under different curing conditions relevant to both laboratory research and practical applications.

#### 2.2.3. Fluidity

The flowability of fresh UHPC slurry was evaluated in accordance with the Chinese standard GB/T 2419-2005 [23]. The test procedure involved the following steps: (1) Mold Preparation: Fresh slurry was poured into a truncated cone-shaped mold (top inner diameter: 70 mm; bottom inner diameter: 100 mm; height: 60 mm) in two successive layers. Each layer was compacted 15 and 10 times, respectively, using a standardized tamping rod. (2) Mold Removal: The mold was carefully lifted vertically upward to ensure minimal disturbance to the slurry. (3) Vibration and Measurement: The slurry underwent vibration for 25 s on a jump table. Subsequently, the maximum spread diameters along two mutually perpendicular directions were measured using a calibrated steel ruler. The flowability of UHPC was determined by averaging these two diameters.

#### 2.2.4. Mechanical Properties

According to Chinese standard GB/T 50081-2019 [24] and T/CBMF 37-2018 [25], flexural test specimens and compressive test specimens were prepared separately according to the requirements, and flexural strength and compressive strength tests were conducted on UHPC specimens, respectively. An electro-hydraulic servo universal testing machine (YAW-3000) was used to test the compressive strength of UHPC specimens. Six standard specimens (sized 40 mm × 40 mm × 40 mm) were prepared for each mix proportion for compressive strength testing, and the arithmetic mean was taken as the representative value. A universal testing machine (UTM5105) was used for the three-point bending test. The flexural strength test adopted the three-point bending method, and three standard specimens (sized 40 mm × 40 mm × 160 mm) were prepared for each mix proportion. The arithmetic mean was taken as the representative value.

#### 2.2.5. Autogenous Shrinkage

According to the testing requirements of ASTM C1698-2009 (2014) [26], non-contact displacement sensors were used to measure the early autogenous shrinkage of UHPC. The test conditions were set as follows: a maintenance environment temperature of (20 ± 2) °C and a relative humidity of (60 ± 5)%. The specific operation was as follows: The sample was prepared according to the reference mix ratio (excluding steel fibers), and a secondary vibration process was used (60 vertical vibrations and 60 horizontal vibrations) to fill the corrugated pipe. After measuring the initial length with a vernier caliper, the specimen was placed in the equilibrium position of the autogenous shrinkage tester, 5 min intervals were set for continuous monitoring, and finally, the autogenous shrinkage time curve was obtained.

#### 2.2.6. Mercury Intrusion Porosimetry

The porosity of UHPC samples was measured using mercury intrusion porosimetry (MIP). The instrument adopted was the Auto Pore TV 9500 mercury intrusion porosimeter. After the compressive strength test, the central part of the test block was soaked in anhydrous ethanol for 7 d, and then dried in a 60 °C oven for 3 d. A block sample of about 3 g crushed to 2–3 mm was taken for testing, with a pore size testing range of 3 nm to 350 μm.

#### 2.2.7. Thermogravimetry Analysis

Thermogravimetric (TG) analysis experiments were performed using a STA449F3 simultaneous thermal analyzer (Netzsch, Selb, Germany). The test parameters were set as follows: the furnace was fed with high-purity nitrogen as a protective gas, the starting temperature was 30 °C, and the temperature was increased to 1000 °C at a rate of 10 °C/min. Based on the synchronous thermal analysis curve (DSC-TG), the calcium hydroxide (Ca(OH)_2_) in the hydration products of the samples was quantitatively characterized.

#### 2.2.8. Scanning Electron Microscope

A model VEGA3 TESCAN (SEM) (Provided by China Taisiken Co., Ltd., Tianjin, China) was used to observe the microscopic morphology of the hydration products and the internal structure of UHPC at a working distance of 11–15 mm, a resolution of 50–400 um, and an accelerating voltage of 15 KV.

## 3. Results and Discussion

### 3.1. Fluidity

**Figure 4:**
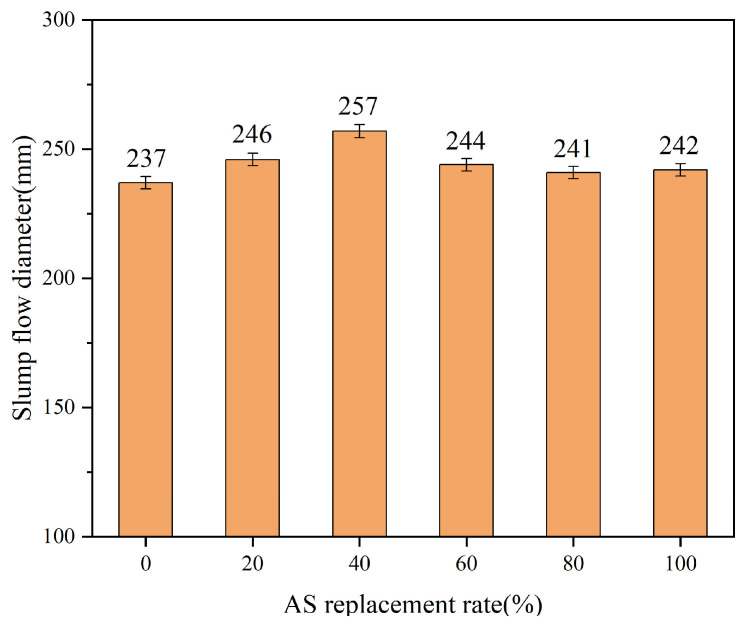
Fluidity of UHPC with different AS replacement rates.

The flowability of fresh UHPC slurries with different AS replacement rates are shown in Figure 4. It can be seen that adding AS instead of river sand as a fine aggregate was beneficial for improving the flowability of fresh UHPC slurry. The flowability of fresh UHPC slurry showed a trend of first increasing and then decreasing with the increase in AS content. The flowability of the freshly mixed UHPC slurry in the blank group was 237 mm. When the replacement rates of AS in the experimental group were 20%, 40%, 60%, 80%, and 100%, the flowability of fresh UHPC slurry was 246 mm, 257 mm, 244 mm, 241 mm, and 242 mm, respectively. Compared with the blank group, the flowability increased by 3.8%, 8.4%, 3.0%, 1.7%, and 2.1%, respectively. When the AS content was 40%, the improvement in flowability of the fresh UHPC slurry was most significant.

This phenomenon can be attributed to the unique morphological characteristics of AS. Compared to RS 1, the AS particles exhibited higher sphericity and smoother surfaces, reducing interparticle friction and promoting a “balling effect” that enhanced slurry mobility. Furthermore, AS filled the macropores between RS 1 particles, liberating the entrapped slurry originally occupying smaller voids. This redistribution increased the effective slurry volume in the fresh mixture, thereby improving fluidity [6]. However, beyond 40% AS substitution, the gradation of fine aggregates became uneven, and the fineness modulus declined. At this threshold, AS particles no longer optimized packing efficiency, leading to reduced fluidity due to agglomeration and disrupted particle distribution.

### 3.2. Mechanical Properties

**Figure 5:**
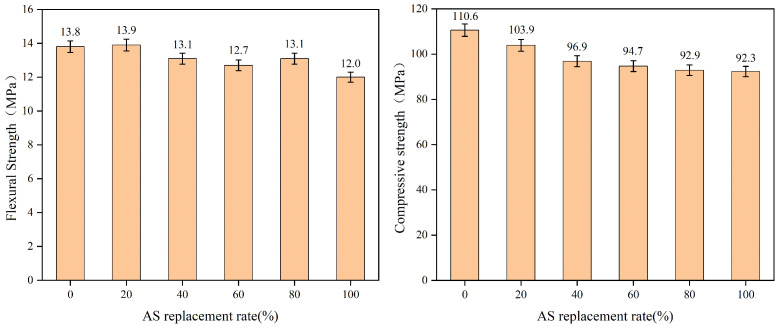
Compressive and flexural strength of UHPC with different AS replacement rates (standard curing for 28 d).

The flexural and compressive strengths of UHPC with varying AS replacement rates (0–100%) under standard curing (28 d) are illustrated in Figure 5. Both properties exhibited a gradual decline with increasing AS content, contrasting with the trends observed in ordinary concrete. For conventional concrete, flexural and compressive strengths typically peak at intermediate AS substitution levels (e.g., 20–60%) due to AS’s ability to fill the voids between coarse aggregates, optimizing gradation and enhancing densification [27,28]. However, in UHPC—composed predominantly of fine particles (RS and powders) without coarse aggregates—AS’s filling effect is limited, resulting in monotonic strength reductions at higher substitution rates.

**Figure 6:**
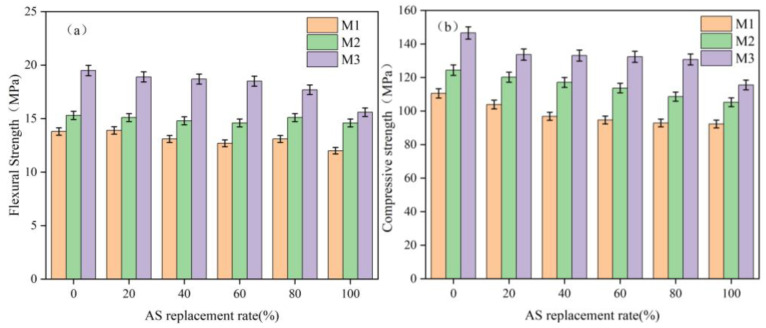
Mechanical properties of UHPC with different AS replacement rates: (**a**) flexural strength, (**b**) compressive strength.

The effects of different curing regimes on the flexural and compressive strength of UHPC with different AS replacement rates are shown in Figure 6. The results showed that the flexural and compressive strengths of the test blocks under curing with steam at 90 °C showed a significant increase compared to those under standard curing. For example, in the group with 60% and 80% AS content, the flexural strengths were 12.7 MPa and 13.1 MPa, while the compressive strengths were 94.7 MPa and 92.9 MPa under the standard curing regime. The flexural strengths reached 14.6 MPa and 15.1 MPa, respectively, under the M2 regime, which were 14.9% and 15.3% higher compared to the M1 regime, and the compressive strengths were 113.7 MPa and 108.6 MPa, which were 20.1% and 16.9% higher than the M1 regime. In the M3 regime, the flexural strengths were 18.5 MPa and 17.7 MPa, which were 45.7% and 35.1% higher than the M1 regime, and the compressive strengths were 132.4 MPa and 130.8 MPa, respectively, which were 39.8% and 40.8% higher than the M1 regime. This enhancement stemmed from steam curing’s activation of pozzolanic reactions. Silica fume (highly reactive amorphous SiO_2_) reacts with Ca(OH)_2_ from cement hydration to form an additional C-S-H gel, refining the pore structures and densifying the matrix [29]. Minor amorphous SiO_2_ in AS further contributed to this reaction under elevated temperatures.

While the highest strengths were achieved with 0% AS replacement, the 60–80% replacement samples still met UHPC strength requirements (>120 MPa compressive strength). This strength reduction was attributed to the following: (1) differences in the particle packing efficiency between AS and RS; (2) the higher water demand of AS particles; and (3) the slightly increased porosity with higher AS content. However, the optimized curing regime (M3) minimized these effects, demonstrating that high AS replacement is feasible with proper processing.

The significant performance differences between curing regimes (M1–M3) highlighted the critical importance of curing protocol selection for AS-UHPC. While standard curing (M1) produced adequate results, the combined steam + standard curing (M3) consistently yielded superior mechanical properties, suggesting that this approach may be optimal for practical applications where both early-age strength and long-term performance are important.

### 3.3. Autogenous Shrinkage

**Figure 7:**
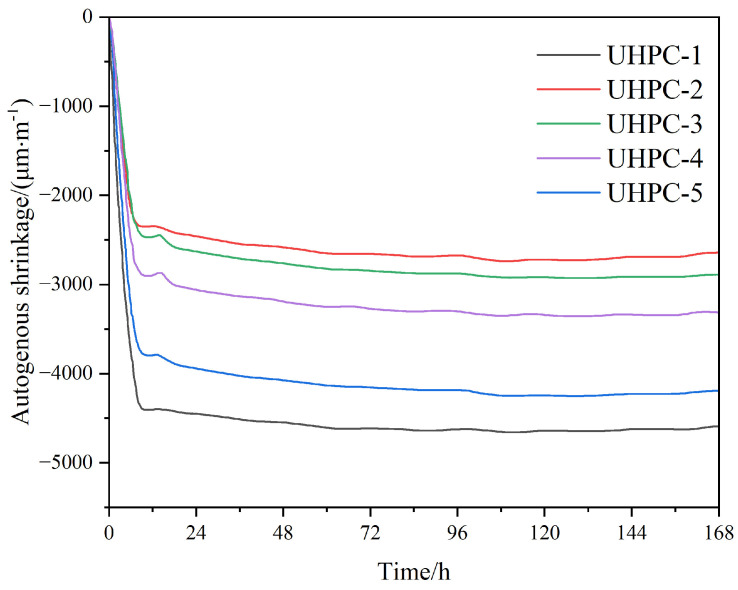
Effects of RS 1 replaced by AS on autogenous shrinkage of UHPC.

Figure 7 shows the trend of the autogenous shrinkage of UHPC with different AS content; all the data are the values of the autogenous shrinkage of UHPC after final setting. From the figure, it can be found that the autogenous shrinkage of UHPC is large, and is several times greater than the shrinkage value of ordinary concrete. At 12 h after final condensation, the autogenous shrinkage of UHPC was 4404, 2345, 2465, 2897, and 3795 μm/m when the content of AS was 0%, 20%, 40%, 60%, and 80%. The autogenous shrinkage showed a trend of first decreasing and then increasing with increasing AS content, and the shrinkage stabilized after 24 h. The shrinkage of UHPC primarily stemmed from capillary tension caused by self-drying during cement hydration. As the AS content increased, the specific surface area of particles per unit volume in UHPC rose significantly. This amplified the water demand for the hydration reaction and slowed the hydration rate. Additionally, a higher AS content refined the pore structures, further increasing capillary tension. Consequently, the internal free water diminished under these combined effects. With the reduced internal free moisture, when the cementitious material was hydrated, the material internal self-drying phenomenon occurred in advance, leading to the relative increase in autogenous shrinkage [19,30]. The non-linear relationship between AS content and shrinkage stemmed from competing mechanisms: at a low AS content (<40%), the spherical AS particles improved packing and reduced shrinkage. At higher contents, the increased surface area led to greater water demand and capillary tension.

It is worth noting that the degree of autogenous shrinkage of UHPC containing AS was generally lower than that of UHPC without AS, which was due to the fact that with the increase in AS content, the gradation of aggregate particles gradually improved and the porosity decreased, which improved the cracking-resistant ability and volume stability of the matrix, and the autogenous shrinkage could be reduced by 13.83% to 34.22% when the content of AS ranged from 60% to 80%. This indicated that the degree of autogenous shrinkage of UHPC with a high AS content was also within the controllable range.

### 3.4. Pore Structure Analysis

The internal pore structure of cementitious materials can be categorized into four types according to the pore size: (0, 10) nm pores are gel pores, [10, 100] nm pores are small capillary pores, (100, 5000] nm pores are large capillary pores, and >5000 nm pores are large pores. Among them, the gel pores with a pore size of less than 10 nm can be regarded as harmless pores, and their influence on the concrete properties can be neglected. The small capillary pores with a pore size of 10–100 nm will significantly affect the properties of concrete materials. The large capillary pores with a pore size of more than 100 nm as well as the macropores are usually regarded as being harmful pores, which will have a serious impact on the mechanical properties and durability of concrete [31].

**Figure 8:**
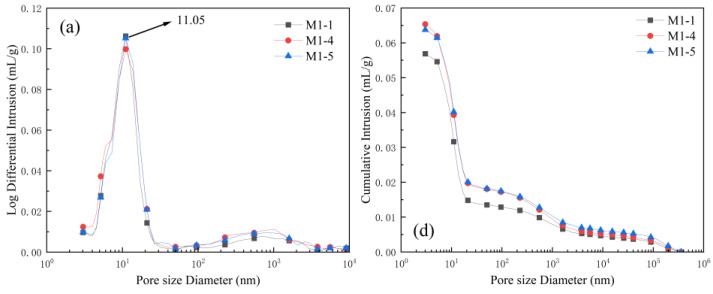

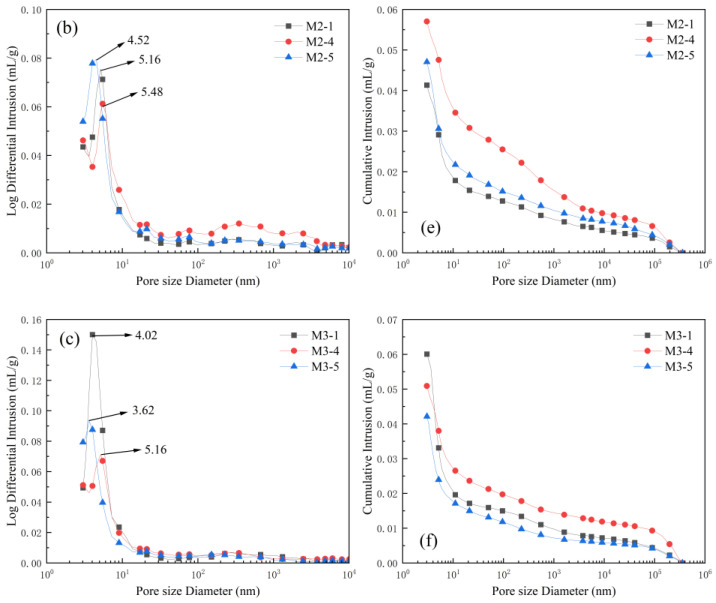
Analysis of pore structure of UHPC under different curing regimes (M1, M2, M3): differential pore volume (**a**–**c**); cumulative pore volume (**d**–**f**).

To evaluate the impact of high AS substitution on pore refinement, UHPC4 (60% AS) and UHPC5 (80% AS) were selected for detailed analysis, as these levels represented the optimal balance between AS utilization (>60%) and retained mechanical integrity under hybrid curing (M3). Table 4 shows the effect of no AS content and 60% and 80% AS content on the porosity of UHPC under different curing regimes. Figure 8 shows the effect of no AS content and 60% and 80% AS content on the pore volume distribution of UHPC under different curing regimes. From Table 4, it can be seen that with the increase in AS content, the porosity decreased. Compared to the M1 and M2 system, the overall porosity of the M3 system decreased. In the same curing system, with the increase in AS content and the gel pore content below 10 nm and above 100 nm, the large capillary pore and macroporous content decreased and increased.

A comprehensive analysis of Table 4 and Figure 8 shows that the main pore size distribution of UHPC under the M1 regime is concentrated to 100 nm and below, with small capillary pores dominating and a low gel pore content, so the compressive strength of UHPC under the M1 regime is lower, and the large number of small pores and the concentrated small capillary pores reduce the mechanical properties of the material. By accelerating the cement hydration process through steam curing, it can clearly be seen that a large number of small capillary pores under the M1 system are further refined, which leads to the distribution of the main pore size of UHPC under the M2 system being concentrated to 10 nm and below, with the proportion of the total porosity accounting for about 50%, and by continuing to carry out curing, it is found that the content of gel pores under the M3 system is further increased, which leads to a significant increase in the compressive strength of UHPC under the M3 system compared to that of M2 and M1. Comparing Table 4 and Figure 8a–c, it can be found that the most available pore size of M1-4 and M1-5 decreases from 11.05 nm to 5.48 nm and 4.52 nm by steam curing, and after steam curing and continuing the standard curing, the most available pore size is further decreased to 5.16 nm and 3.62 nm, and the main pore size distribution is changed to gel pores, which indicates that the combination of steam and standard curing has a significant effect on the UHPC mechanical properties and durability performance [32].

The main reasons for the above changes in pore structure are as follows: (1) The particle size of AS is smaller than the particle size of RS 1, and by replacing RS 1 with an equal mass, the porosity of the UHPC decreases. (2) The water–gel ratio used in the preparation of the UHPC is low, and with the increase in the content of the AS, the specific surface of the particles per unit volume of the UHPC increases dramatically, which leads to an increase in the amount of water needed for the hydration reaction of the concrete, and so the hydration rate slows down and the concrete cannot be generated. More C-S-H gel fills the aggregate pores and refines the pore structure, resulting in a decrease in the gel pore content [33]. (3) Steam curing to accelerate the UHPC cement hydration process, and silica fume, which contains a large number of reactive SiO_2_ and has a strong volcanic ash activity, in the process of the second hydration will generate more C-S-H gel to fill the pore space. This will further refine the pore structure, resulting in a substantial increase in the pore content of the gel, which in turn improves the mechanical properties and durability of UHPC [34,35].

### 3.5. TG-DTG Analysis

**Figure 9:**
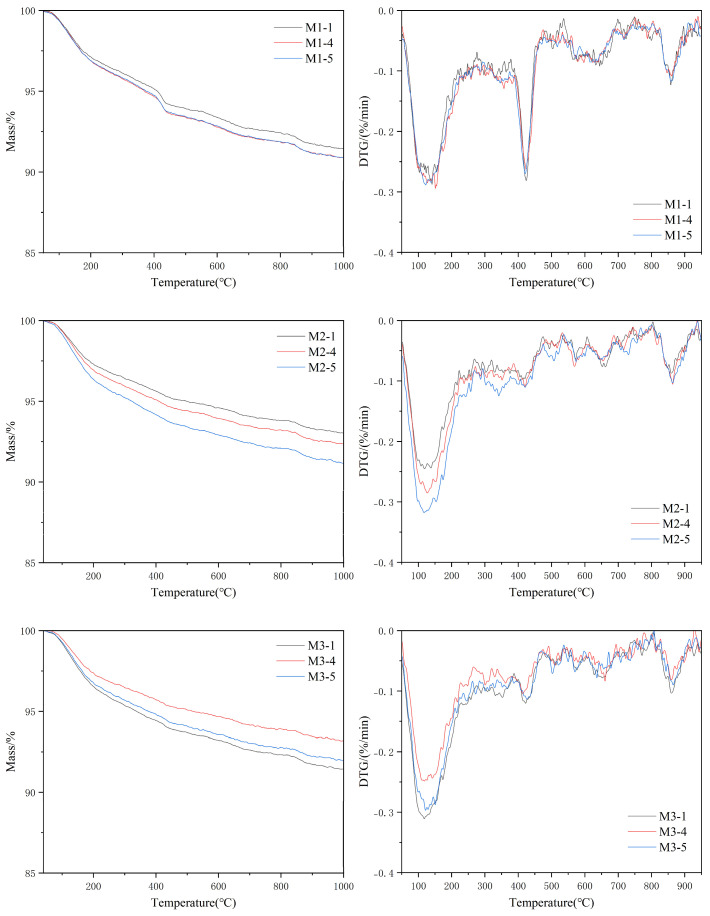
TG and DTG under M1 system, M2 system, and M3 system.

The thermogravimetric (TG) and derivative (DTG) curves of the three AS substitution rates of UHPC in the M1, M2, and M3 regimes are shown in Figure 9. From the graphs, it is initially concluded that the incorporation of AS leads to a reduction in the final residual mass. In the DTG curves, the mass loss at around 100 °C was caused by the evaporation of free water, and the mass loss at around 400 °C was caused by the dehydration of the C-S-H gel and Ca(OH)_2_ crystals [36,37]. Ca(OH)_2_ decomposed at around 420 °C, and based on the DTG curves, the temperature range at which the decomposition of Ca(OH)_2_ occurred was considered to be from 380 to 450 °C. It was noteworthy that the Ca(OH)_2_-induced mass loss peak at about 420 °C was significantly lower in the specimens after steam curing compared to the specimens after 28 d of standard curing.

The Ca(OH)_2_ content in each sample at each conditioning regime can be calculated according to the following formula, as shown in Equation (3):

(3) $$W_{CH}=-\frac{M_{CH}m_{420}}{M_{H_{2}O}m_{800}}$$

where W_CH_ is the Ca(OH)_2_ content, M_CH_ and M_H_2_O_ are the molar mass of Ca(OH)_2_ and water, and m_420_ and m_800_ are the mass loss at around 420 °C (380 °C to 450 °C) and the residual mass obtained at 800 °C.

The calculated Ca(OH)_2_ contents of the UHPC samples with a 0%, 60%, and 80% replacement rate of AS in the M1 regime were 9.58%, 10.15%, and 9.91%, which suggests that AS does not have the property of promoting secondary hydration or participating in secondary hydration under the ambient conditions of standard curing. The mass loss in the interval from 105 °C to 540 °C was caused by the amount of non-evaporated water, which was 6.01%, 6.66%, and 6.54% for the three groups. Under the same curing regime and a fixed water content (Table 2), the incorporation of AS increased the amount of non-evaporated water. While total water remained constant, AS’s higher specific surface area altered the distribution and availability of water within the matrix. The finer AS particles increased the interfacial area between the solids and water, enhancing water adsorption on particle surfaces and reducing free water mobility. This localized water retention prolonged the hydration kinetics, allowing for more water to participate in pozzolanic reactions over time, despite the fixed total water content. This mechanism improved the utilization of water for secondary hydration (e.g., SiO_2_–Ca(OH)_2_ reactions), thereby influencing the mechanical and durability properties [38,39].

The Ca(OH)_2_ contents of UHPC samples with 0%, 60%, and 80% AS substitution rates under the M2 regime were 6.45%, 7.03%, and 8.59%. This indicates that steam curing at 90 °C significantly promotes the consumption of Ca(OH)_2_ in the AS-UHPC. The Ca(OH)_2_ contents of the UHPC samples with 0%, 60%, and 80% AS substitution rates under the M3 regime were 4.77%, 6.62%, and 6.87%. The Ca(OH)_2_ content showed minimal variation between the M3 and M2 curing regimes. This indicates that steam curing predominantly drives AS-UHPC’s secondary hydration and pozzolanic reactions during the early stages. Prolonging the curing time post-steam treatment, however, does not significantly enhance further AS hydration. Instead, extended curing primarily facilitates the continued consumption of Ca(OH)_2_ and promotes silica fume’s pozzolanic reaction. These combined processes ultimately improve UHPC’s strength and durability properties [40].

### 3.6. SEM Analysis

**Figure 10:**
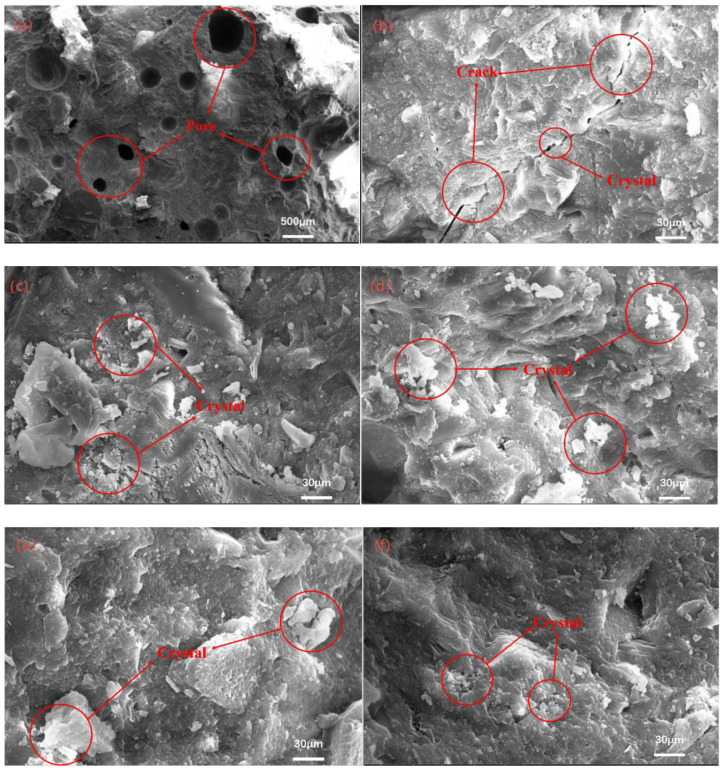
SEM of UHPC with 60% replacement rate of AS under M1 system (**a**,**b**), M2 system (**c**,**d**), M3 system (**e**,**f**). (Red circle in the picture: Calcium crystals or microcracks).

The SEM images of the 60% AS substitution rate UHPC at 28 d of standard curing are shown in Figure 10a,b. It can be observed that for a larger number of pore pores, the pore size is defined as being a small capillary pore or larger, there are very obvious cracks between the aggregate and slurry, there are more cracks in the matrix, the hydration product is not sufficiently dense, and from the figure, it can be observed that there are plate-like calcium hydroxide crystals at the interface, which are very poorly bonded to the interface [41]. This is because the water requirement of the AS is large, the hydration of cement is not sufficient in the early stage, and the slurry area with a high water–cement ratio is formed around the AS, which makes it easy for calcium hydroxide to accumulate [42,43].

SEM images of UHPC with a 60% AS substitution rate under the M2 curing regime are shown in Figure 10c,d. At this time, the high crystallinity of the overall structure, the dense accumulation of crystal products, and the increased structural defects at the interface have not changed, and many pores and cracks can be observed in the gel, as well as a small amount of layered calcium hydroxide crystal accumulation. The interface of the aggregate and the slurry is also more tightly bonded, which can be seen in the autoclaving conditions. It can be seen that under the vaporization conditions, the activity is highly exerted and the microstructure of UHPC is relatively dense [44].

SEM images of UHPC with a 60% AS substitution rate under the M3 curing regime are shown in Figure 10e,f. After steam curing and continuing to undergo a period of standard curing, it can be observed that the number of cracks in the matrix is reduced, but they still exist, which is caused by temperature stress; the interface between the aggregate and the matrix is further optimized, with a tight bond at the interface; calcium hydroxide crystals and more pronounced cracks are not observed; and the hydration product accumulation is dense. The subsequent hydration structure plays a role in filling and perfecting, indicating that the curing method can improve the replacement rate of AS and improve the strength of the UHPC matrix [45].

## 4. Conclusions

This study investigates the feasibility of replacing RS with AS as an aggregate in UHPC, especially at substitution rates of 60% to 80%. This research found that optimized particle gradation significantly enhanced the flowability and density of UHPC. The experimental results demonstrated that appropriate steam and standard curing conditions not only improved the compressive and flexural strength of the concrete but also effectively reduced early autogenous shrinkage and lowered the porosity. Steam curing accelerated the hydration of cement and silica fume, facilitating the formation of additional C-S-H gel. This process densified the microstructure, thereby improving the mechanical properties and durability. These findings confirm that AS serves as an effective and environmentally beneficial substitute for a UHPC aggregate. The main conclusions are summarized as follows:

(1) A high-density UHPC skeleton was generated using the modified Andreasen and Andersen particle filling model. The deviation of the particle size distribution between the target and mixture curves was small. The combination of the grading of AS and RS with cementitious materials was favorable for obtaining the optimal particle size distribution profile of UHPC.
(2) Under the standard curing regime, UHPC with 60% to 80% AS incorporation increased the degree of flow by 1.7% to 3.0% and reduced the degree of autogenous shrinkage by 13.83% to 34.22% as compared to the blank group. A significant reduction in the flexural and compressive strength of UHPC was observed under the standard 28 d curing regime.
(3) Under the M3 curing regime (steam curing at 90 °C for 2 d followed by standard curing for up to 7 d), UHPC with 60–80% AS substitution achieved a flexural strength exceeding 18 MPa and a compressive strength over 130 MPa. Notably, the compressive strength of samples with 60% and 80% AS reached 132.4 MPa and 130.8 MPa, respectively. Compared to standard 28 d curing, this hybrid regime reduced porosity by 31.7% (from 13.4% to 9.2%) and increased the gel pore content by nearly 1.5 times (from 23–27% to 58–61%), demonstrating superior matrix densification through accelerated hydration and pozzolanic reactions. These results highlight the feasibility of high-volume AS utilization in UHPC while maintaining exceptional mechanical properties.

This paper mainly studies the early- and mid-term performance of AS-UHPC. In the future, further research can be conducted on its long-term performance, especially its durability performance under different environmental conditions (such as freeze–thaw cycles, wet–dry alternation, etc.), to evaluate the potential application of AS-UHPC in practical engineering.

## Figures and Tables

**Table 1:** Oxide composition of cement, slag, and silica fume/(mass %).

Substance	Cement	Slag	SF
CaO	62.05	38.80	1.25
SiO_2_	21.94	32.27	94.44
Al_2_O_3_	5.82	16.17	0.35
Fe_2_O_3_	2.63	0.41	0.14
K_2_O	0.67	0.56	0.37
Na_2_O	0.19	0.46	0.19
SO_3_	2.09	2.67	0.12
MgO	2.82	7.25	0.56
TiO_2_	0.30	0.77	0.10
MnO	0.09	0.20	0.01
P_2_O_5_	0.06	-	0.39
Cl^−^	0.10	0.03	0.05
Other	1.24	0.41	2.04

**Table 2:** Design of UHPC mix AS/(kg/m^3^) proportions.

Group	Cement	SF	Slag	RS 2	RS 1	AS	Water	SP	LSF (Vol.%)
UHPC-1	774.7	166.0	166.0	169.3	959.6	0	210.3	22.1	0.65
UHPC-2	774.7	166.0	166.0	169.3	767.7	191.9	210.3	22.1	0.65
UHPC-3	774.7	166.0	166.0	169.3	575.7	383.8	210.3	22.1	0.65
UHPC-4	774.7	166.0	166.0	169.3	383.8	575.7	210.3	22.1	0.65
UHPC-5	774.7	166.0	166.0	169.3	191.9	767.7	210.3	22.1	0.65
UHPC-6	774.7	166.0	166.0	169.3	0	959.6	210.3	22.1	0.65

**Table 3:** Abbreviation annotations and meanings.

Sample Code	AS Substitution Rate	Curing Regime	Description
UHPC-1–UHPC-6	0–100%	M1, M2, or M3	See Table 2 for mix proportions; curing regimes defined in Section 2.2.2
M1	N/A	Standard (20 °C, 28 d)	Control curing condition
M2	N/A	Steam (90 °C, 2 d)	High-temperature accelerated curing
M3	N/A	Steam (2 d) + standard curing (5 d)	Hybrid curing for optimized hydration

**Table 4:** Porosity of UHPC.

Specimen	Porosity/%	Aperture Distribution/%			
		(0, 10) nm	[10, 100] nm	(100, 5000] nm	>5000 nm
M1-1	12.24	28	50	13	9
M1-4	13.72	27	47	17	9
M1-5	13.43	23	50	17	10
M2-1	12.15	54	16	15	15
M2-4	11.94	52	16	14	18
M2-5	10.24	52	16	15	17
M3-1	12.08	66	10	12	12
M3-4	10.87	61	13	12	14
M3-5	9.17	58	14	14	14

Note: M1: Standard curing (28 d); M2: Steam curing (2 d); M3: Steam (2 d) + standard curing (5 d). Numeric suffixes (e.g., −1, −4, −5) denote AS substitution rates (0%, 60%, 80%) as per Table 2.

## Data Availability

The original contributions presented in this study are included in the article. Further inquiries can be directed to the corresponding author.

## Abbreviations

The following abbreviations are used in this manuscript:

UHPC	Ultra-high-performance concrete
AS	Aeolian sand
RS	River sand
